# Editorial: Kinase/phosphatase signaling and axonal function in health and disease

**DOI:** 10.3389/fncel.2023.1172836

**Published:** 2023-03-24

**Authors:** Gerardo Morfini, Stefan Kins

**Affiliations:** ^1^Department of Anatomy and Cell Biology, University of Illinois at Chicago, Chicago, IL, United States; ^2^Division of Human Biology and Human Genetics, University of Kaiserslautern - Landau (RPTU), Kaiserslautern, Germany

**Keywords:** kinase, phosphatase, axon, phosphorylation, SARS-CoV-2, mammalian target of rapamycin, SARM1, PP2A

Axons represent the main cellular specialization supporting the output of information from neurons. These long cytoplasm extensions provide a physical conduit for electrical signals to propagate from the somato-dendritic compartment of neurons to their target cell(s) and for the bidirectional exchange of trophic information between these cells. Depending on the specific neuronal subtype, human axons extend over distances that range from a few microns to over a meter in length. Accordingly, axons contain proportions of the neuronal cytoplasm that far surpass that of the somato-dendritic domain by thousands of orders of magnitude. Remarkably, nearly all cellular components contained in axons must be actively transported from their main site of synthesis at the neuronal soma. This daunting cellular process, collectively referred to as *axonal transport* (AT) (Black, 2016), is further complicated because depending on their length and degree of arborization, axons can feature thousands of discrete subcompartments of unique biochemical compositions (Matsuda et al., 2009). The large size and complex subcellular architecture of axons are typically underrepresented in most schematic drawings of neurons, including the ones depicted in Figure 1.

**Figure 1:**
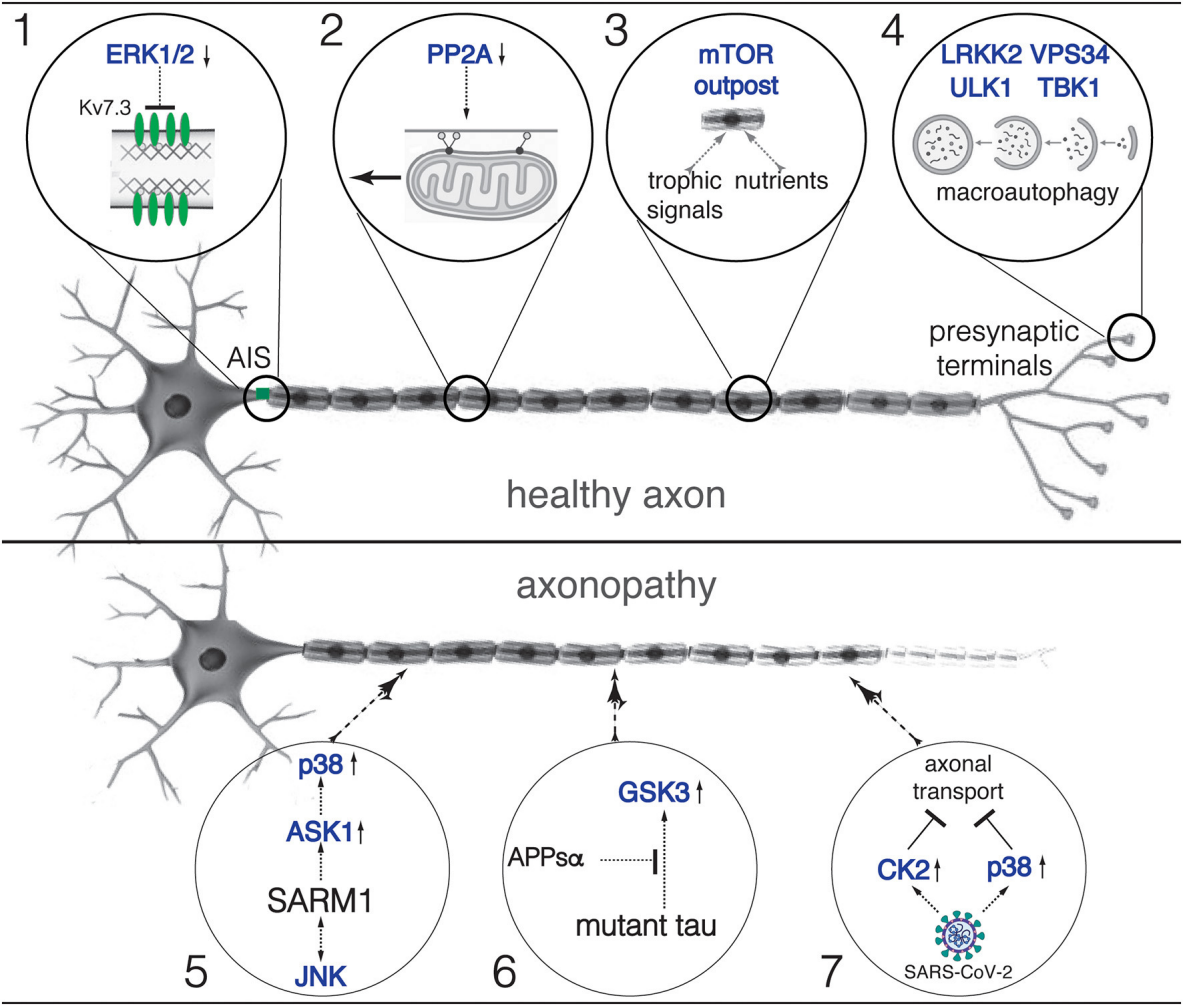
Roles of specific protein kinases, phosphatases and phosphorylation-based mechanisms on axonal function and pathology. Schematic representation of mature motor neurons bearing a healthy **(top)** and a degenerating axon **(bottom)**. Circles 1 to 7 provide a succinct summary of kinases, phosphatases (both indicated by text in blue), and phosphorylation-based mechanism addressed by each manuscript in this Research Topic [1: see Baculis et al.; 2: see Heo et al.; 3: see Altas et al.; 4: see Berth et al.; 5: see Waller and Collins; 6: see Baltissen et al.; 7: see Richards and Jaesnich]. Small arrows pointing up and down indicate heightened and reduced kinase/phosphatase activities, respectively. Inhibitory effects are indicated by blunt arrows. Within each circle, dashed lines indicate that specific effects might be indirect.

The axonal initial segment (AIS), nodes of Ranvier, and presynaptic terminals are primary examples of discrete axonal subcompartments sustaining neuronal connectivity. Their specialized functions require a continuous, highly localized supply and turnover of *unique complements* of membrane-bounded organelles (MBOs), cytoskeletal and soluble protein components. For example, the rapid propagation of action potentials in myelinated neurons depends on acute voltage-sensitive portions of the axonal plasma membrane at the AIS and nodes of Ranvier. This is achieved by the local insertion of vesicles containing specific subsets of voltage-gated sodium and potassium channels at the AIS axolemma, as well as the insertion of vesicles containing a different subset of channels to the nodes of Ranvier's axolemma (Huang and Rasband, 2018). Similarly, proteins involved in the release and recycling of synaptic vesicles are selectively delivered at the presynaptic terminal's plasma membrane (Watson et al., 2023). These observations have long-implied mechanisms for the spatial regulation of cellular processes within axons, including the delivery of selected MBOs to their correct destinations (Morfini et al., 2001).

The correct functionality of specialized axonal subcompartments requires the coordination of diverse cellular processes including AT, assembled and localized remodeling of the cytoskeleton, spatial restriction of signaling pathways, and recycling of old materials and defective MBOs, among many others. From various mechanisms that regulate proteins involved in the execution of these cellular processes, *phosphorylation* represents the most widespread and better-documented mechanism. By extension, research addressing the roles of *kinases* and *phosphatases* in the axonal compartment is essential for a complete understanding of neuronal function.

In this Research Topic, data from two manuscripts support the notion that kinases and phosphatases regulate the motility and delivery of selected MBOs at specific axonal subcompartments. Using pharmacological experiments in cultured hippocampal neurons, a report by Baculis et al. suggests a potential mechanism linking neuronal activity to ERK1/2 kinases and levels of potassium Kv7.3 channels inserted at the AIS (Figure 1.1). In addition, a research article by Heo et al. reveals PP2A as a protein phosphatase involved in the regulation of mitochondrial transport (Figure 1.2). This finding, which involved the use of an innovative, high-throughput screening system based on high-content imaging, is consistent with prior works revealing phosphorylation-dependent regulation of motor proteins powering AT (Gibbs et al., 2015; Morfini et al., 2016).

Two manuscripts discuss both hypothesized and established roles of specific protein kinases in the regulation of cellular processes sustaining axonal health. Altas et al. propose a model where the protein kinase mTOR, a well-established hub for various signaling pathways, accumulates in local axonal foci termed “mTOR outposts”. Such outposts would work as spatial gatekeepers of mTOR-activating stimuli (e.g., nutrients and trophic factors) to collectively modulate neuron-wide responses, including transduction of signals to the distant neuronal nuclei and the control of protein synthesis (Figure 1.3). In addition, a mini-review by Berth et al. discusses published work on the roles played by several protein kinases on specific molecular events supporting macroautophagy, a cellular process involving sequestration, packaging, and delivery of old and defective cellular components to lysosomes for degradation (Figure 1.4).

Consistent with a critical role of kinases and phosphatases on axonal function, a large body of genetic and experimental evidence has linked alterations in their activities to axonal pathology, an early pathological signature common to most neurodegenerative disorders. Specifically, work from various experimental systems revealed that specific neuropathogenic proteins promote abnormal activation of selected axonal kinases and phosphatases, alterations in AT, and axonopathy (Brady and Morfini, 2017). Three manuscripts in this Research Topic relate to this important issue.

A mini-review by Waller and Collins focuses on SARM1, an enzyme that acts as a sensor of metabolic stress and a critical component of pathways leading to axonal degeneration. These authors discuss findings supporting bi-directional regulation of axonal SARM1 and JNK kinases, as well as SARM1's ability to inhibit regeneration of injured axons through activation of the MAPK kinase ASK1 and its downstream effector kinase p38 (Figure 1.5). In addition, a research article by Baltissen et al. reports that a soluble fragment derived from proteolytic cleavage of the Alzheimer's disease-related protein APP ameliorates neuropathological features in a mouse model of a human tauopathy. Interestingly, this beneficial effect was associated with the inhibition of the kinase GSK3, which is aberrantly activated in this model and has been shown to inhibit anterograde AT (Morfini et al., 2002, 2004; Figure 1.6). Finally, an opinion article by Richards and Jaesnich hypothesizes a potential mechanism underlying axonal pathology and neurological complications associated with SARS-CoV-2 infection. This hypothesis is based on independent lines of experimental evidence showing that SARS-CoV-2 promotes the secretion of glial cytokines known to activate the kinases p38 and CK2, and reports show that active forms of these kinases inhibit AT by directly phosphorylating motor proteins (Morfini et al., 2013; Leo et al., 2017; Figure 1.7).

The focus of this Research Topic is unique because it specifically features research on kinases, phosphatases, and phosphorylation-dephosphorylation-based mechanisms relevant to axonal function and pathology. Considering the rapid advance of methodologies for the study of kinases and phosphatases (White and Wolf-Yadlin, 2016), the development of various approaches for the identification of their substrates (Allen et al., 2007), and the availability of experimental models that facilitate the study of molecular events in axons (Kang et al., 2016; Song et al., 2016; Wang et al., 2020), we anticipate the publication of more Special Editions with a similar focus in the near future.

## Author contributions

GM wrote the original draft. Both authors made substantial, direct, and intellectual contributions to the work. Both authors approved the final version of this Editorial for publication.
